# Integrating and Evaluating the Data Quality and Utility of Smart Pump Information in Detecting Medication Administration Errors: Evaluation Study

**DOI:** 10.2196/19774

**Published:** 2020-09-02

**Authors:** Yizhao Ni, Todd Lingren, Hannah Huth, Kristen Timmons, Krisin Melton, Eric Kirkendall

**Affiliations:** 1 Division of Biomedical Informatics Cincinnati Children's Hospital Medical Center Cincinnati, OH United States; 2 Department of Pediatrics University of Cincinnati College of Medicine Cincinnati, OH United States; 3 Wake Forest Center for Healthcare Innovation Wake Forest School of Medicine Winston Salem, NC United States; 4 Indiana University Bloomington, IN United States; 5 Division of Neonatology and Pulmonary Biology Cincinnati Children's Hospital Medical Center Cincinnati, OH United States; 6 Department of Pediatrics, Wake Forest School of Medicine Winston Salem, NC United States

**Keywords:** medication administration errors, smart infusion pumps, electronic health records, concordance

## Abstract

**Background:**

At present, electronic health records (EHRs) are the central focus of clinical informatics given their role as the primary source of clinical data. Despite their granularity, the EHR data heavily rely on manual input and are prone to human errors. Many other sources of data exist in the clinical setting, including digital medical devices such as smart infusion pumps. When incorporated with prescribing data from EHRs, smart pump records (SPRs) are capable of shedding light on actions that take place during the medication use process. However, harmoniz-ing the 2 sources is hindered by multiple technical challenges, and the data quality and utility of SPRs have not been fully realized.

**Objective:**

This study aims to evaluate the quality and utility of SPRs incorporated with EHR data in detecting medication administration errors. Our overarching hypothesis is that SPRs would contribute unique information in the med-ication use process, enabling more comprehensive detection of discrepancies and potential errors in medication administration.

**Methods:**

We evaluated the medication use process of 9 high-risk medications for patients admitted to the neonatal inten-sive care unit during a 1-year period. An automated algorithm was developed to align SPRs with their medica-tion orders in the EHRs using patient ID, medication name, and timestamp. The aligned data were manually re-viewed by a clinical research coordinator and 2 pediatric physicians to identify discrepancies in medication ad-ministration. The data quality of SPRs was assessed with the proportion of information that was linked to valid EHR orders. To evaluate their utility, we compared the frequency and severity of discrepancies captured by the SPR and EHR data, respectively. A novel concordance assessment was also developed to understand the detec-tion power and capabilities of SPR and EHR data.

**Results:**

Approximately 70% of the SPRs contained valid patient IDs and medication names, making them feasible for data integration. After combining the 2 sources, the investigative team reviewed 2307 medication orders with 10,575 medication administration records (MARs) and 23,397 SPRs. A total of 321 MAR and 682 SPR dis-crepancies were identified, with vasopressors showing the highest discrepancy rates, followed by narcotics and total parenteral nutrition. Compared with EHR MARs, substantial dosing discrepancies were more commonly detectable using the SPRs. The concordance analysis showed little overlap between MAR and SPR discrepan-cies, with most discrepancies captured by the SPR data.

**Conclusions:**

We integrated smart infusion pump information with EHR data to analyze the most error-prone phases of the medication lifecycle. The findings suggested that SPRs could be a more reliable data source for medication error detection. Ultimately, it is imperative to integrate SPR information with EHR data to fully detect and mitigate medication administration errors in the clinical setting.

## Introduction

### Background

Electronic health records (EHRs) are the central focus of many efforts in clinical research and quality improvement given their role as the primary clinical data source [1-4]. Despite their granularity, the data heavily rely on manual input and are prone to human errors [3,4]. Many digital devices have been used in clinical environments, and they provide additional sources of data for understanding health care processes, a form of real-world data from clinical settings. One example is digital medication infusion pumps, more commonly known as *smart pumps*. These pumps, which are now commonplace in modern health care settings, record copious amounts of rich, granular data about medication administration. Smart pumps have been shown to prevent some errors while propagating others. One systematic review found that smart pumps can intercept multiple error types, such as wrong dose and wrong rate errors, as well as reduce adverse drug events [5]. This effect, however, is heavily dependent on user compliance and utilization of specific functionalities vendor products afford, including dose error reduction software [6]. As with EHRs, infusion pump alerts are another source of alert burden and are subject to alert fatigue, which raises a trade-off between potential safety benefits and increased workload for providers [7]. Smart pumps, compared with their analogue counterparts, generate a lot of data to log user interaction with the pumps (eg, pausing of pump infusions and pump alert overrides) and the pump status (eg, infusion start and infusion complete), which are associated with granular timestamps. The data create useful information such as user compliance with alerts, pump states at different time points, and mechanical alarm records. These smart pump records (SPRs) can be harnessed to help understand actions that take place during medication administration.

The ability to link and leverage different data sources across the full medication lifecycle has the potential to make medication errors recognizable and rectifiable. Theoretically, when combining smart infusion pump information with prescribing data from EHRs, one can see the bookends of the medication use process, from medication origin (order) to terminus (administration). Although there are gaps in the intermediate steps (traditionally the transcription and dispensing stages), given that errors are more frequent in the ordering and administration phases [8], integrating the EHR and SPR data permits visibility in the most error-prone phases. As such, the harmonization of these 2 data sources can provide insight and information about safe and unsafe practices.

Our research is specifically directed at developing accurate and scalable informatics technologies to monitor the medication use process and detect medication administration errors. In our previous studies, we developed artificial intelligence–based algorithms for monitoring the use of high-risk medications including vasopressors, narcotics, insulin, total parenteral nutrition (TPN), and fluids [1,9,10]. By analyzing order and medication administration record (MAR) data residing in EHRs, the algorithms identified discrepancies and potential errors in how medications were being ordered and documented as administered. Despite their viability in discrepancy detection, the algorithms relied on a single data source that resulted in a number of false positives and false negatives. For instance, the algorithms might miss an error in administration (a false negative) if an order adjustment was not placed in the EHR or was incorrectly documented in the MAR [10]. To improve the accuracy of error detection, we sought to integrate smart pump information into the computerized algorithms.

Integration of SPRs with EHR data requires advanced informatics technologies and is not without significant challenges [4]. Most health care institutions that use smart pumps have not fully integrated them into a *closed-loop* system, which would permit automatic linkage of order data in the EHRs to administration information from the pumps. One barrier to integration is the cost and complexity of the implementation process, including its impact on clinical workflows. Another barrier is the maturity of the technology and its associated challenges [11]. Single-site studies have reported increased work efficiencies and revenue benefits, but widespread integration is not yet ubiquitous [12]. As such, although great potential exists, the insight gained from combining the data has not yet been realized.

### Objectives

To fill these gaps in knowledge, we integrated SPRs with EHR data and evaluated their quality and utility in detecting medication administration errors. Our overarching hypothesis was that SPRs would contribute unique information in the medication use process, enabling more comprehensive detection of discrepancies and potential errors in medication administration. The specific aims of this study were to (1) develop an automated algorithm that aligns SPRs with EHR data to facilitate manual review of medication administration, (2) characterize discrepancies identified from EHRs and SPRs, and (3) develop a novel assessment that measures the concordance between the ability of EHR and SPR data in detecting medication administration discrepancies. This study is among the first to integrate multiple clinical data sources to understand medication safety events. Our long-term objective is to establish a more effective and generalizable program that assembles comprehensive data sources in clinical environments to improve patient safety across health care institutions.

## Methods

### Setting and Study Population

We evaluated medication administration for patients admitted to the neonatal intensive care unit (NICU) at the Cincinnati Children’s Hospital Medical Center (CCHMC). Approval for this study was provided by the CCHMC institutional review board (study ID: 2015-3824), and a waiver of consent was authorized.

The CCHMC NICU is a level 4 NICU that provides the highest level of neonatal intensive care to complex and critically ill newborns. The unit has an average daily census of 70 patients and an average of 750 admissions per year. The institution utilizes a fully computerized commercial EHR system (Epic Systems Corporation). Additional NICU safety interventions include the use of computerized provider order entry with embedded clinical decision support, a bar code medication administration (BCMA) system, smart infusion pump technology with a customized neonatal library of medications, daily prescription review by dedicated NICU pharmacists, and clinical guidelines for high-risk medications.

### Study Medications and Study Periods

We focused on reconciling 9 high-risk, continuous intravenous infusion medications prescribed to NICU inpatients, including vasopressors (dopamine, dobutamine, epinephrine, milrinone, and vasopressin), narcotics (fentanyl and morphine), TPN, and lipids. Continuous intravenous infusions have a higher risk and severity of error than other medication administrations [13,14]. In particular, its administration usually spans multiple nursing shifts and involves complex dosage adjustments that are not captured by in-place interventions such as BCMA. Medication administrations for vasopressors and narcotics were studied over the period of January 1, 2014, to December 31, 2014. Due to changes to our ordering system, TPN and lipid administrations were studied over the period of January 1, 2016, to December 31, 2016. All vasopressors, narcotics, and lipid orders were included in the analysis. Owing to the large volume of TPN orders, we randomly selected 8.05% (669/8308) of the TPN orders for analysis.

### Clinical Data Extraction and Federation

Medication use information was extracted retrospectively from the institution’s EHR system. The information included (1) medication orders that documented infusion doses (or infusion rates) prescribed to the patients, (2) structured order modifications that adjusted the original doses and rates via computerized physician order entry, (3) MARs documented by clinical professionals that describe doses or rates administered to patients, and (4) free-text physician to nurse communication orders that specified complex medication dose or rate adjustments during patient care. The infusion pump information was extracted separately from the vendor-provided reporting system (CareFusion). The information included (1) patient IDs, (2) medication names, and (3) SPRs that documented actual doses or rates administered to patients. The SPRs contained multiple pump state categories including infusion started or restarted, stopped, completed, paused, canceled, and delayed. Only SPRs that indicated infusion started or restarted were used for this analysis because they represented the initiation of medication delivery and the point at which one would want to intercept potential erroneous infusions.

As the SPRs were not integrated into the institution’s EHR system, there was no explicit association between an SPR and its corresponding medication order. As such, we developed a computerized algorithm to merge the 2 data sources and align SPRs with their potential medication orders. The EHR and SPR data were first grouped by patient IDs and medication names. The algorithm then chronologically aligned the EHR and SPR data for each patient medication group, where each SPR was linked to the closest medication order with order placement, modification, or MAR documented within 24 hours of its administration. The SPRs with invalid patient IDs or unknown medication names could not be definitely linked to any order. As such, they were excluded from subsequent manual review and discrepancy analysis.

### Manual Review for Gold Standard Creation

**Figure 1:**
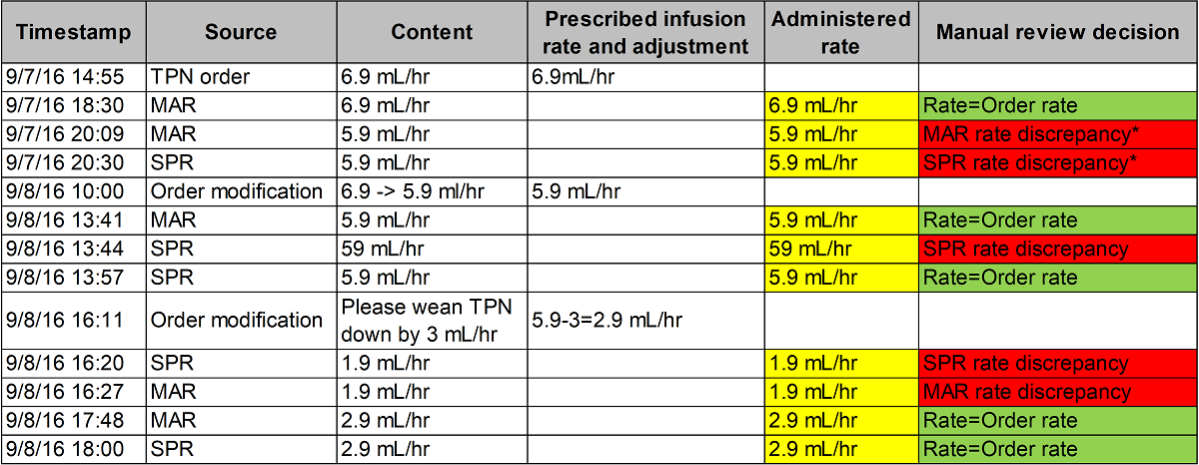
An example of chronological ordering of medication use data and medication administration records or smart pump record discrepancies identified by manual review. The discrepancy occurred because the order modification was placed over 30 min after the MAR or SPR, which did not meet the institutional expectations even if the administration was correct. MAR: medication administration records; SPR: smart pump record; TPN: total par-enteral nutrition.

A clinical research coordinator (CRC) and 2 board-certified pediatric physicians on the research team (including 1 neonatologist) manually reviewed the aligned data for each patient medication group to identify medication administration discrepancies in MARs and SPRs. Figure 1 illustrates an example of the chronological ordering of EHR and SPR data and discrepancies identified by manual review. A discrepancy was defined as a mismatch between the prescribed dose or rate of a medication and the electronic documentation of its administration in MARs or SPRs [10]. A discrepancy may be a medication administration error, or it may be a false positive subject to further investigation. For the purposes of this study, we defined an a priori 30-min window to allow for verbal orders to be transcribed into the EHR, in line with our institutional policy and expectations. As such, a discrepancy occurred if an order was placed more than 30 min after an administration, even if the correct dose or rate was administered (the starred discrepancy in Figure 1). If a discrepancy was detected, the reviewers additionally identified the correct dose or rate prescribed. Differences between the reviewers’ decisions were resolved during the adjudication sessions. Inter-rater reliability was calculated using Cohen kappa to define the agreement [15].

### Analysis of Discrepancies

The consensus annotations served as a gold standard to understand medication use processes and discrepancies. To assess the data quality, we analyzed the proportion of valid information in the SPR source. An SPR was valid if it contained both a valid patient ID and a medication name. An ID was considered valid if its value mapped to an existing patient ID in the NICU. Clinical staff manually entered patient IDs into the infusion pumps; hence, invalid IDs may represent entry or programming errors. Medication names were present in the SPRs if the staff selected their profile from the pump drug library. Infusions programmed under a generic *basic* infusion status did not have a medication associated with the records. We then investigated the number of discrepancies identified by MARs and SPRs to characterize the scale of discrepancies captured by the 2 sources. The magnitude of discrepancy (MoD), as defined by the percentage of discrepancy over a correct dose or rate, was also calculated to quantify the severity of a discrepancy. Finally, we developed a concordance assessment to understand the detection power and capabilities of the MAR data alone, the SPR data alone, and their overlap. We hypothesized that MAR discrepancies often represented documentation errors. As such, the use of a concordance measure could help differentiate documentation issues versus true administration errors, reflected by the concordance of the MAR and SPR discrepancies or SPR discrepancies alone. The assessment first divided each order sequence into multiple event blocks separated by order modifications (either order initiations or modifications and audits). It then identified whether an event block contained 1 of 4 categories: no discrepancies, MAR-only discrepancies, SPR-only discrepancies, or both MAR and SPR discrepancies. For example, the TPN order in Figure 1 contained 3 event blocks, 1 with an SPR-only discrepancy and 2 with both MAR and SPR discrepancies. Medication orders containing both MARs and SPRs were included in the analysis. The descriptive statistics of the 4 categories were calculated for each medication and in aggregation to study the concordance. Cohen kappa was calculated to assess the agreement between MAR and SPR discrepancies.

## Results

Table 1 presents the distribution of SPRs with and without valid patient IDs and medication names in the SPR data source. A total of 543,791 out of 764,624 SPRs (71.11%) in 2014 and 521,113 out of 787,692 SPRs (66.16%) in 2016 contained valid patient IDs and medication names and were therefore feasible for data federation. Of the 220833 invalid SPRs in 2014, 66.7% (147,304) were because of invalid patient IDs, 52,680 (23.%) were because of missing medication names, and 20,849 (9.4%) were because of missing identifiers. A similar distribution of invalid SPRs was observed in the 2016 data. Table 2 shows the distribution and categorization of valid and invalid patient IDs documented in the SPRs.

**Table 1:** The distribution of smart pump records with and without valid patient IDs and medication names.

Data sources		Patient ID+^a^, n (%)	Patient ID−^b^, n (%)	Total, n (%)
**2014 data**				
	Medication name+	543,791 (71.12)	147,304 (19.26)	691,095 (90.38)
	Medication name−	52,680 (6.89)	20,849 (2.73)	73,529 (9.62)
	Total	596,471 (78.01)	168,153 (21.99)	764,624 (100.00)
**2016 data**				
	Medication name+	521,113 (66.16)	175,830 (22.32)	696,943 (88.48)
	Medication name−	63,326 (8.04)	27,423 (3.48)	90,749 (11.52)
	Total	584,439 (74.20)	203,253 (25.80)	787,692 (100.00)

^a^The patient ID or medication name was valid.

^b^The patient ID or medication name was invalid or missing.

**Table 2:** Descriptive statistics of patient IDs in smart pump records and categorization of invalid patient IDs.

Groups		Year	
		2014	2016
Valid patient IDs, n		569	476
**Invalid patient IDs, n**		173	148
	Date of birth out of range (1965-2014), n (%)	42 (24.3)	25 (16.9)
	Entering patient names instead of IDs, n (%)	33 (19.1)	10 (6.8)
	Missing digits in patient IDs, n (%)	30 (17.2)	23 (15.5)
	Random numbers, n (%)	23 (13.3)	10 (6.8)
	Entering encounter IDs instead of patient IDs, n (%)	20 (11.6)	49 (33.1)
	Invalid letters in patient IDs, n (%)	13 (7.5)	8 (5.4)
	Expired patient IDs due to merged charts, n (%)	4 (2.3)	10 (6.8)
	Extra digits in patient IDs, n (%)	4 (2.3)	10 (6.8)
	Potential typographical errors in patient IDs, n (%)	4 (2.3)	3 (2.0)

Table 3 presents the descriptive statistics of the medication use data. The CRC and physicians reviewed 2307 medication orders with 10,575 MARs and 23,397 SPRs during the study period. A total of 321 discrepancies were identified from MARs (discrepancy rate 321/10,575, 3.0%), and 682 discrepancies were identified from SPRs (discrepancy rate 682/23,397, 2.9%). The overall inter-rater reliabilities were 0.92/0.90 (MAR/SPR), indicating almost perfect agreement on decision making [15]. Among the targeted medications, vasopressors including epinephrine, dopamine, and vasopressin had the highest discrepancy rates, followed by narcotics (fentanyl) and TPN. The SPR discrepancy rates were higher than that of MARs for all medications except epinephrine.

**Table 3:** Descriptive statistics of the gold standard medication use data.

Medication	Patients, n (%)	Orders, n (%)	MAR^a^, n (%)	MAR discrepancy, n (% total^b^)	MAR discrepancy rate, %	SPRs^c^, n (%)	SPR discrepancy, n (% total)	SPR discrepancy rate, %
Dobutamine	1 (0.2)	3 (0.1)	7 (0.1)	0 (0.0)	0.0	18 (0.1)	0 (0.0)	0.0
Dopamine	10 (1.6)	18 (0.8)	60 (0.6)	4 (1.2)	6.7	152 (0.6)	13 (1.9)	8.6
Epinephrine	87 (13.6)	325 (14.1)	1937 (18.3)	233 (72.6)	12.0	2994 (12.8)	290 (42.5)	9.7
Fentanyl	38 (6.0)	134 (5.8)	723 (6.8)	9 (2.8)	1.2	2332 (10.0)	87 (12.8)	3.7
Lipid	179 (28.1)	604 (26.2)	1725 (16.3)	0 (0.0)	0.0	1884 (8.1)	6 (0.9)	0.3
Milrinone	33 (5.2)	71 (3.1)	744 (7.0)	3 (0.9)	0.4	1188 (5.1)	14 (2.1)	1.2
Morphine	110 (17.2)	434 (18.8)	2850 (27.0)	13 (4.0)	0.5	10,051 (43.0)	150 (22.0)	1.5
TPN^d^	160 (25.1)	669 (29.0)	2281 (21.6)	43 (13.4)	1.9	4524 (19.3)	105 (15.4)	2.3
Vasopressin	20 (3.1)	49 (2.1)	248 (2.3)	16 (5.0)	6.5	254 (1.1)	17 (2.5)	6.7
Overall	638 (100.0)	2307 (100.0)	10,575 (100.0)	321 (100.0)	3.0	23,397 (100.0)	682 (100.0)	2.9

^a^MAR: medication administration record.

^b^The numbers in parentheses represent the percentage of total discrepancies attributable to a medication.

^c^SPR: smart pump record.

^d^TPN: total parenteral nutrition.

**Figure 2:**
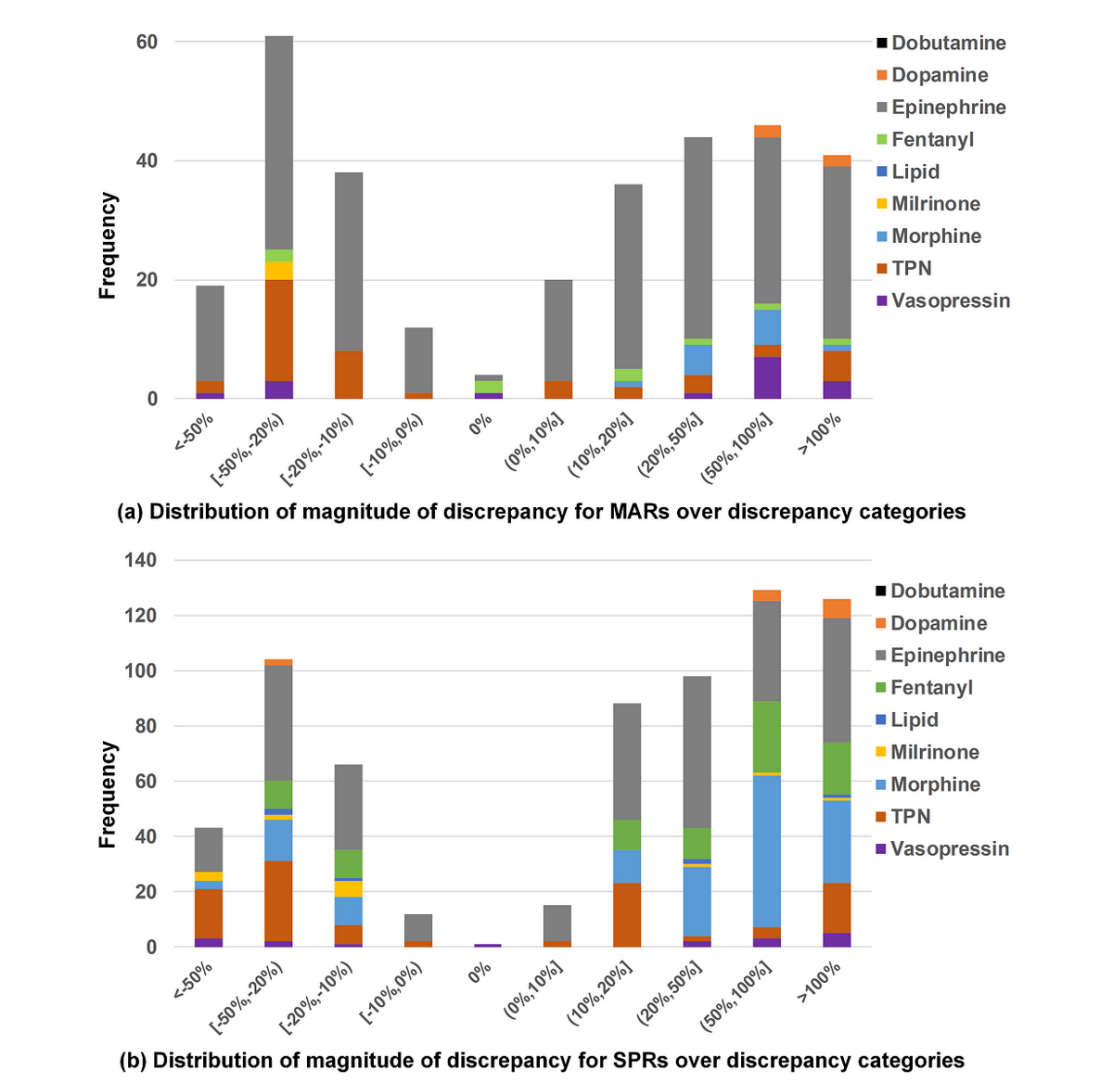
Distribution of magnitude of discrepancy for (a) medication administration records and (b) smart pump records over discrepancy categories. MARs: medication administration records; SPRs: smart pump records; TPN: total parenteral nutrition.

**Figure 3:**
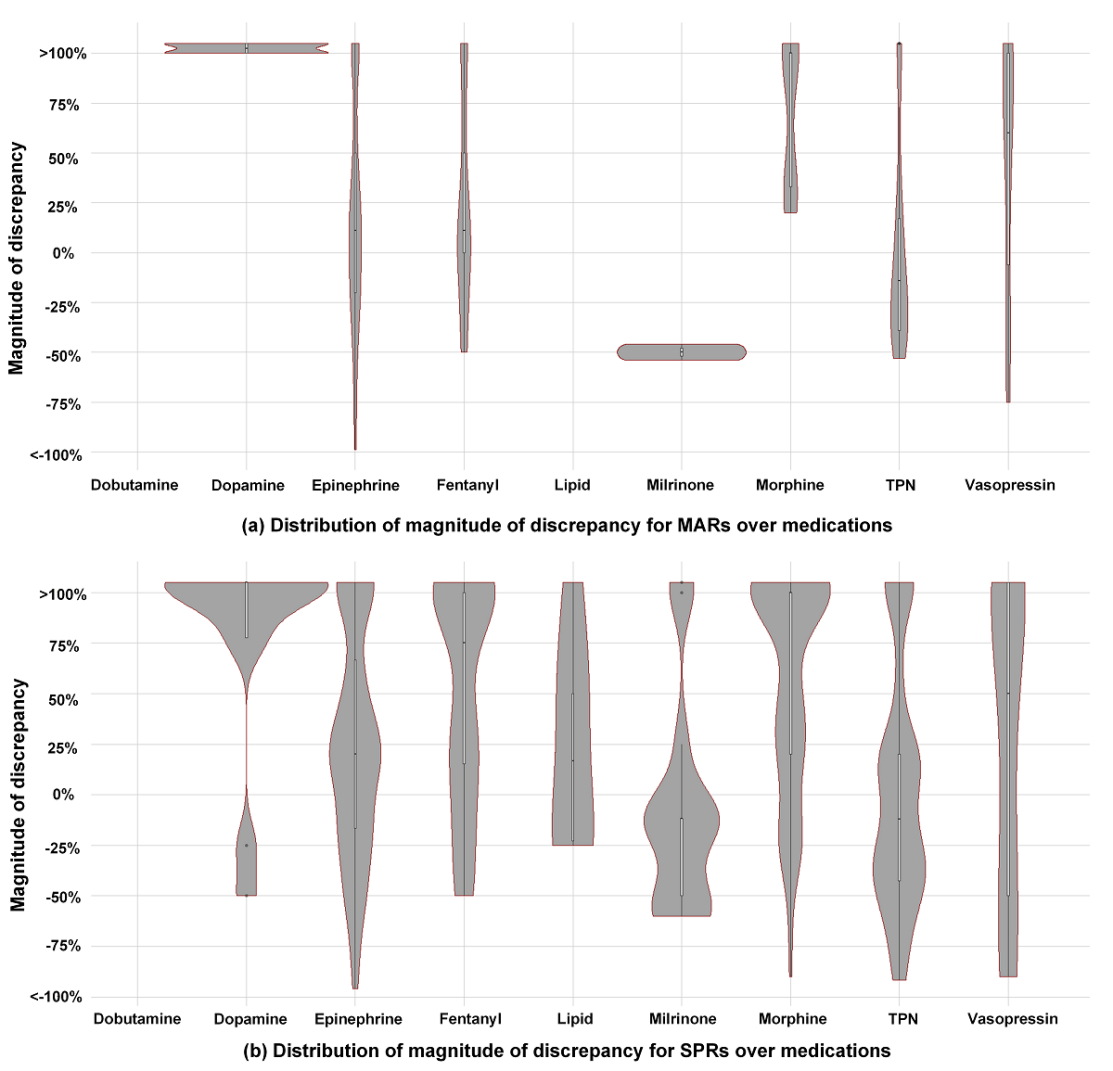
Distribution of magnitude of discrepancy for (a) medication administration records and (b) smart pump records over medications. MARs: medication administration records; SPRs: smart pump records; TPN: total parenteral nutrition.

Table 4 presents the MoD for MARs and SPRs across all targeted medications. A total of 58.2% (187/321) of the MAR discrepancies were overdoses, of which 21.9% (41/187) were substantial overdoses (administered dose was 100% greater than the prescribed dose). A total of 66.9% (456/682) of the SPR discrepancies were overdoses, of which 27.6% (126/456) were substantial overdoses. The few discrepancies with 0% magnitude represent documentation issues where the administrated doses or rates were correct but the orders or order modifications were placed more than 30 min after administration. Figure 2 depicts the MoD distributions for MARs and SPRs over discrepancy categories. Epinephrine, fentanyl, morphine, and TPN were responsible for most MAR and SPR discrepancies, particularly for substantial overdoses. Figure 3 depicts the MoD distributions for MARs and SPRs for each medication. Dopamine, epinephrine, and vasopressin showed similar distributions between MARs and SPRs. Other medications such as fentanyl, morphine, and TPN had low numbers of substantial overdoses on MARs but higher numbers on SPRs.

**Table 4:** Magnitude of discrepancy for medication administration records and smart pump records across all medications.

Data sources	Magnitude of discrepancy, n (%)									
	<−50%	[−50%,−20%)	[−20%,−10%)	[−10%,0%)	0%	(0%,10%]	(10%,20%]	(20%,50%]	(50%,100%]	>100%
MAR^a^	19 (5.9)	61 (19.0)	38 (11.8)	12 (3.7)	4 (1.2)	20 (6.2)	36 (11.2)	44 (13.7)	46 (14.3)	41 (12.8)
SPR^b^	43 (6.3)	104 (15.2)	66 (9.7)	12 (1.8)	1 (0.1)	15 (2.2)	88 (12.9)	98 (14.4)	129 (18.9)	126 (18.5)

^a^MAR: medication administration record.

^b^SPR: smart pump record.

Table 5 presents the concordance between MAR and SPR discrepancies. The analysis included 60.58% (1397/2306) medication orders that contained both MARs and SPRs. The orders were segmented into 2638 event blocks, of which 308 (11.67%) had discrepancies. Of these 308 blocks, 197 (64.0%) contained only SPR discrepancies, 44 (14.3%) contained only MAR discrepancies, and 67 (21.7%) contained both. The Cohen kappa was 0.32, suggesting minimal agreement between MAR and SPR discrepancies [15]. The event blocks with SPR discrepancies were higher than those with MAR discrepancies across all targeted medications.

**Table 5:** Concordance assessment between medication administration record and smart pump record discrepancies.

Medications	Orders^a^, n		Analysis block^b^, n	MAR^c^ discrepancy^d^, n	SPR^e^ discrepancy^d^, n	Concordance category, n			
	All	Included				None	MAR	SPR	Both
Dobutamine	3	2	3	0	0	3	0	0	0
Dopamine	17	12	40	4	13	35	1	3	1
Epinephrine	325	189	901	196	288	793	25	37	46
Fentanyl	134	82	182	6	79	145	3	32	2
Lipid	604	352	353	0	6	349	0	4	0
Milrinone	71	42	60	1	14	51	1	8	0
Morphine	434	315	631	12	143	529	5	91	6
TPN^f^	669	380	380	27	105	347	8	15	10
Vasopressin	49	23	88	4	17	78	1	7	2
Overall	2306	1397	2638	250	665	2330	44	197	67

^a^All represents the orders in the data set, whereas *Included* represents the orders included in the concordance analysis (ie, the orders having both MAR and SPR data associated with them).

^b^Analysis block represents the event blocks included in the analysis.

^c^MAR: medication administration record.

^d^MAR discrepancy and SPR discrepancy represent the MAR or SPR discrepancies, respectively, found in the analysis blocks and included in the analysis.

^e^SPR: smart pump record.

^f^TPN: total parenteral nutrition.

## Discussion

### Principal Findings

This study is among the first to integrate smart infusion pump information with EHR data to analyze the most error-prone phases of the medication use process, recognizing that linkage of complex data has its challenges [4]. Smart pump data lack clinical usefulness without appropriate identification of both patient information and medication being used at the time of infusion. One of the main findings was that 29%-34% of the smart pump data were not valid because of missing patient or medication information (Table 1). Most of these missing data were because of invalid patient information, which was caused by mistakes during ID entry on the pumps or unfamiliarity with documentation guidelines during infusion pump programming (Table 2). A large portion of invalid IDs was because of misentries such as missing or adding extra digits, invalid letters, and typographical errors. In addition, a patient ID might be replaced with the patient name or encounter ID, suggesting a mistake in following the documentation guidelines or a workaround. Missing medication information occurred when a basic infusion was selected without specifying the medication being administered. This occurs most commonly as a workaround when the correct medication cannot be found in the smart pump library. When patient or medication identifiers are incorrect or missing, linking smart pump data with order logs or MARs, particularly in real time, becomes vastly more complicated and unreliable. Inference by time of administration is difficult because commonly administered medications (eg, TPN) might have been concurrently ordered for several patients in the same unit.

We identified higher SPR discrepancies than MAR discrepancies (Table 3), suggesting that SPRs could be a more reliable source of error detection than EHR data. This finding also implies that the frequency of medication errors reported in the literature might be underestimated when limited to analysis of EHR data alone [1,9,10]. Both MARs in EHRs and smart pump programming rely on manual data entry and are prone to human error. For example, bar code scanning inputs MAR data into the EHR based on medication label information, but clinical staff must validate the dose, which may change as medications are titrated. Similarly, without a closed-loop system where the pump is automatically programmed by an order, smart pump programming also relies on human data entry. However, compared with MARs, smart pump entries are closest to a patient, representing the truest reflection of what the patient receives. The SPR discrepancies we identified may represent different types of errors. They may be secondary to unintentional misprogramming (ie, the nurse programs an incorrect rate or drug concentration) or misunderstanding (ie, the nurse does not understand an order or misses an order modification), but we are unable to determine the exact causes in this study using only retrospective data. Further studies should investigate the distribution of error types for MARs and SPRs and discuss the effectiveness of corresponding error prevention strategies.

Not all discrepancies have clinical significance, and for most medications, very small discrepancies are not as important as large ones. As observed in our studies, minor discrepancies are typically more numerous (Table 4) [10]. Other studies have also noted that medication errors are numerous but are often small and associated with low rates of harm [7,16-19]. The risk of calling out these frequent, small discrepancies is an increase in workload and decrease in overall attention. It is widely known, for example, that EHR alerts that identify frequent events are perceived as *noisy* (ie, providing erroneous or irrelevant information) and are overridden at high rates [20,21]. As such, we measured the MoDs to assess their severity. Although the analysis demonstrated many minor results, we also detected a notable amount of substantial dosing discrepancies (Table 4). In particular, discrepancies in substantial dosing were dominated by certain medications (eg, epinephrine; Figure 2) and were more commonly detectable from SPR data (Figure 3). These findings suggest the necessity of integrating SPR data into medication error detection, which informs further development of our real-time notification system for medication error events [10]. Imagining a future where multiple data sources are incorporated to detect medication errors in real time, one can see the benefit that the MoD holds. The assessment conveys to a provider not just that an error event has occurred but also the severity of the event to guide his or her clinical response.

Recognizing that SPR and MAR discrepancies may occur together or individually, we developed an assessment to measure their concordance in the same medication cycle (Table 5). Although there were limitations to this methodology, as we had to limit the analysis to only 60.5% (1397/2306) of orders containing both MARs and SPRs, the use of the concordance assessment allowed us to separate documentation issues (MAR-only discrepancies) from true discrepancies (SPR-only discrepancies and matched MAR or SPR discrepancies). Over 85% of the discrepancies were captured by SPRs, implying that the majority were true discrepancies. This trend was consistent across all targeted medications. The kappa statistics suggested that there was little overlap between MAR and SPR discrepancies, and only 21.7% of the discrepancies were captured by both data sources. These novel findings again indicated the necessity of incorporating SPRs into understanding the medication use process. They make smart pump data more clinically and safety relevant, connecting to our ultimate goal of repurposing clinical data to improve the quality of clinical care.

Given that medication discrepancies occur with relative frequency, efforts to improve smart pump use must continue. Several studies have demonstrated the importance of continuous quality improvement with regular assessment of smart pump data [11,12]. Methods that require less device programming, such as the use of barcode scanning pumps, may help reduce pump programming and patient identification errors. Although closed-loop systems have been implemented in several institutions as another method to address these concerns, there have been issues with titration of medications and specific error types remain unmitigated [22-24]. As such, efforts must focus on ways to utilize medication use information from smart pumps to recognize and address errors as quickly as possible. Our ongoing work focuses on incorporating smart pump data into a real-time error notification system and developing new approaches to visualize the medication use process as a means to help frontline clinical providers utilize and learn from the information at hand. By integrating data from multiple sources, we will move medication error detection systems from retrospective and reactive to prospectively preventive and proactive.

### Limitations

There are limitations to this study. First, although we were able to utilize approximately 70% of the data, excluding SPRs with missing patient and medication information may have resulted in data bias. Efforts have been initiated to improve the data quality of SPRs via quality improvement. Second, the institution’s IT infrastructure does not allow for the delivery of real-time smart pump information, limiting us to medication discrepancy detection and not intervention. Consequently, we must categorize identified events as discrepancies, as we lacked the real-time clinical information to define them as errors. To mitigate this issue, we will increase the frequency of SPR review to a daily basis to capture more real-time information. Third, we chose to focus our work on high-risk continuous medications, and the rates of discrepancy and smart pump issues may differ for intermittent medications. In addition, the study focused on detecting discrepancies in medication doses or rates, which did not capture other error types such as prematurely stopping medications. Future work has been initiated to extend our analysis for intermittent medications and other types of medication use errors. Finally, our data reflected ordering practices and smart pump utilization in a single intensive care unit at a single institution. To assess the generalizability of our findings, project planning and communication are in progress to implement the study in an adult health care institution.

### Conclusions

In this study, we integrated smart infusion pump information with EHR data to analyze the most error-prone phases of the medication lifecycle. We identified more discrepancies from SPRs compared with EHR MARs. The MoD assessment also demonstrated that substantial dosing discrepancies were more commonly detectable from SPRs. The concordance analysis showed little overlap between MAR and SPR discrepancies, with most discrepancies captured by the SPR data. The findings suggested that SPRs could be a more reliable data source for medication error detection. Ultimately, it is imperative to integrate SPR information with EHR data to fully detect and mitigate medication administration errors in the clinical setting.

## Abbreviations

BCMA: bar code medication administration
CCHMC: Cincinnati Children’s Hospital Medical Center
CRC: clinical research coordinator
EHR: electronic health record
MAR: medication administration record
MoD: magnitude of discrepancy
NICU: neonatal intensive care unit
SPR: smart pump record
TPN: total parenteral nutrition
